# Medical College and Hospital Notes

**Published:** 1898-08

**Authors:** 


### MEDICAL COLLEGE AND HOSPITAL NOTES.

The Fresh Air Mission Hospital, at Athol Springs, opened the first week in July for the season. It is a free Hospital for cholera infantum and other diarrheal diseases. Railroad tickets and cards of admission may be obtained at the Fitch Institute at any time. There are eight trains each way daily and suitable cases can be sent promptly without any delay or red tape. The mother must accompany the child if possible.

The hospital is only ten miles from Buffalo and can be reached by train in less than half an hour. Physicians of the city who feel interested to visit the hospital are urged to do so. It is a place practically perfect for its work, both in equipment and in situation. The excursion is a pleasant one for a summer afternoon, and if any physicians who wish to go will telephone the Fitch Institute they can receive tickets at half price, that is, for 25 cents for the round trip.

The hospital means more than giving pleasure; it is a matter of life and death and it is fortunate that this department will not have to be stopped at all this summer. The resident physicians at the hospital this summer will be Dr. Roemmelt and Dr. Filsinger. Miss Galbraith will be the head nurse. The staff physicians, Dr. Dewitt H. Sherman and Dr. Irving M. Snow, make daily visits from Buffalo.

---

THE American Medical Association at its recent meeting unanimously adopted the following:

WHEREAS, the American Medical Association did, at Detroit in 1892, unanimously resolve to demand of all the medical colleges of the United States the adoption and observance of a standard of requirements of all candidates for the degree of doctor of medicine which should in no manner fall below the minimum standard of the Association of American Medical Colleges, and

WHEREAS, this demand was sent officially by the permanent secretary to the deans of every medical college in the United States and to every medical journal in the United States, now therefore the American Medical Association gives notice that hereafter no professor or other teacher in, nor any graduate of, any medical college in the United States, which shall after January 1, 1899, confer the degree of doctor of medicine or receive such degree on any conditions below the published standard of the Association of the American Medical Colleges, [shall] be allowed to register as either delegate or permanent member of this association.

*Resolved*, that the permanent secretary shall, within thirty days after this meeting, send a certified copy of these resolutions to the dean of each medical college in the United States and to each medical journal in the United States.

